# Gender and Acceptance of E-Learning: A Multi-Group Analysis Based on a Structural Equation Model among College Students in Chile and Spain

**DOI:** 10.1371/journal.pone.0140460

**Published:** 2015-10-14

**Authors:** Patricio E. Ramírez-Correa, Jorge Arenas-Gaitán, F. Javier Rondán-Cataluña

**Affiliations:** 1 Escuela de Ingeniería, Universidad Católica del Norte, Coquimbo, Chile; 2 Departamento de Administración de Empresas y Marketing, Universidad de Sevilla, Sevilla, España; University of Toronto, CANADA

## Abstract

The scope of this study was to evaluate whether the adoption of e-learning in two universities, and in particular, the relationship between the perception of external control and perceived ease of use, is different because of gender differences. The study was carried out with participating students in two different universities, one in Chile and one in Spain. The Technology Acceptance Model was used as a theoretical framework for the study. A multi-group analysis method in partial least squares was employed to relate differences between groups. The four main conclusions of the study are: (1) a version of the Technology Acceptance Model has been successfully used to explain the process of adoption of e-learning at an undergraduate level of study; (2) the finding of a strong and significant relationship between perception of external control and perception of ease of use of the e-learning platform; (3) a significant relationship between perceived enjoyment and perceived ease of use and between results demonstrability and perceived usefulness is found; (4) the study indicates a few statistically significant differences between males and females when adopting an e-learning platform, according to the tested model.

## Introduction

E-learning is a fast-spreading way for students to participate in their courses. In fact, students enrolled in one or more online courses is increasing ten times faster than new enrolments in undergraduate education [1, 2].

The development of e-Learning is mainly explained by two factors: (1) the competitive cost advantage, (2) the facilitating qualities such as enhanced reach and learning impact [3]. The purpose of this growth is to supplement traditional instruction, making it possible to develop methods for more portable and flexible learning [4]. According to this idea, many universities around the world have used e-learning environments in the last decade, to the point that educational technologies have become an integral part of the teaching-learning process in tertiary education [5]. But the growth and easy access to information and communication technology is vastly changing the way e-learning courses are conducted, especially in undergraduate education, due to the providing institutions being the most connected Internet communities [1].

Previous studies identified the relevance of individual aspects in influencing the acceptance of eLearning [6]. Furthermore, in learning platforms where the information is reaching critical amounts, the user demands a more personalised and adaptive system interaction [7], and perhaps gender would be one of the individual variables to use for discriminating information.

In terms of Web technology, women and men differ in their levels of trust, risk aversion and information processing, but also in their attitudes and instrumental motives of using and accepting Web environments [8]. In addition, some studies indicated that females communicated more, have a greater social presence, and are more satisfied with online courses than males [9, 10]. Nowadays, we live in the information and communication era, but, are there still differences between males and females with regard to the acceptance and use of e-learning? In this sense, we propose that the diagnostic perceived usefulness (the degree to which using a technology will provide benefits to individuals in performing certain activities) and perceived ease of use (the degree of simplicity associated with individuals’ use of technology) of e-learning environments must be a first step towards correcting possible deviations and promoting the appropriate use of these constructs in university teaching. In turn, results may help the proper design of such environments to respond to the different motivations of students.

In a broader context, gender is considered a cornerstone to explain inequalities and identities in modern society [11]. Against the background of the adoption of information technologies, and particularly from the theoretical perspective of the Technology Acceptance Model, the literature recognises that gender is a key element to understand the differences in perceptions of usefulness and ease of use as determinants of technology adoption [12]. But with regard to e-learning platforms, does gender affect how college students adopt information technology to provide efficient and effective learning solutions?

The main aim of this study is to evaluate whether the adoption of e-learning in two universities, and in particular, the relationship between the perception of external control and perceived ease of use, is different because of gender differences. In order to achieve this goal, a review of the literature on e-learning platforms and Technology Acceptance Model is developed. Based on this review, a research model based on Technology Acceptance Model is proposed to measure the acceptance and use of e-learning by respondents. Third, the results of applying partial least squares analysis to the research model of the entire sample, and the sub-samples of women and men are presented. A t-test was used to analyse if gender differences exist in the different constructs of the model, and a partial least squares multi-group test was utilised to examine differences between groups. Finally, the main conclusions are exposed.

### E-Learning in higher education

E-Learning is defined as an Internet-enabled learning process [13]. This type of learning is causing students to become more autonomous with respect to the teacher and bringing about a significant move from teacher-centred training to student-centred self-regulated learning [14].

Some studies indicate that courses which operate solely in an e-learning context have a higher dropout rate than their face-to-face counterparts [15]. E-learning should be adapted to many circumstances and several approaches to adjustment have been used [16].

For instructors and learners, the consequences of e-learning are widespread. However, in the case of trainers, some studies have not found significant differences between the teacher performance in online teaching and in face-to-face teaching [17]. If these differences do exist, they are likely due to the instructor’s involvement with and the institution’s commitment to the deployment of the method of instruction. Another inference of virtual learning is the intensification of international rivalry for undergraduates by many educational institutions. New communication methods are useful tools that encourage the internationalisation of tertiary learning [18]. Additionally, e-learning reproduces the innovative go-ahead answer to the requirements of an information society and suggests autonomy and impartiality to be able to reach knowledge outside the understanding of cultural and social restrictions [19]. On the other hand, the notion of gender differences has fascinated people for years, and in general it has been believed that these differences are large and immutable. While gender differences have been reported in relation to learning (especially in verbal and mathematical abilities) in the past, now some studies suggest that these differences remain only in some areas. Therefore, it is necessary to investigate whether there are gender differences in the use or perception of e-learning. If gender differences exist, it will be necessary to implement integration policies with regard to these technologies by college managers.

### E-Learning and Technology Acceptance Model

Among the models that have been proposed for understanding the user adoption and usage of information technology innovations, Technology Acceptance Model is one of most widely adopted and tested across organisational contexts, technologies and cultures [20]. The original Technology Acceptance Model [21] postulates that the user’s attitude (the positive or negative feeling of an individual about performing the intention behaviour) towards using the system is determined by its perceived usefulness and its perceived ease of use. Also, perceived usefulness is directly impacted by perceived ease of use. Moreover, behavioural intention (the degree to which a person has formulated conscious plans to perform or not perform some specified future behaviour) to use the system is determined by the user’s attitude towards using the system and users’ perceived usefulness. Finally, behavioural intention determines the actual use of the system. User’s attitude was removed from the Technology Acceptance Model at a later stage because it was felt that attitude was not significantly linked to technology usage [20]. External factors might be important determinants in order to more accurately evaluate the adoption of information technology [21]. Regarding this subject, several revisions and expansions have developed the original model. The most popular developments have been Technology Acceptance Model 2 [22] and Technology Acceptance Model 3 [12]. Technology Acceptance Model 2 extended the original model to explain perceived usefulness and behavioural intention in terms of social influence and cognitive instrumental processes. Technology Acceptance Model 3 prolonged the original model to explain perceived ease of use through the anchoring and alteration framing of individual decision-making.

Technology Acceptance Model can be extended to the e-learning context [23]. According to this, several articles apply Technology Acceptance Model to evaluate users’ acceptance of e-learning technology. In these academic studies the target populations have been university students, teachers, and workers.

In most of these articles, Technology Acceptance Model was prolonged using predictor constructs. Subjective norms (the extent to which individuals perceive that other important people believe that they should use a particular technology) are an important construct explicating the use of an e-learning platform [24–27]. The perception of external control (the extent to which an individual believes that an organisational and technical infrastructure exists to support the use of the system) has shown a significant effect on perceived ease of use [28]. Both perceived usefulness and perceived ease of use of e-learning are influenced by computer self-efficacy (the extent to which an individual believes that he or she has the ability to execute a specific task/job using a computer) [26, 29]. This has also had a positive effect on the behavioural intention [27, 30]. Perceived playfulness (the position of cognitive spontaneity in computer interactions) affects both perceived usefulness and perceived ease of use, and perceived playfulness directly affects behavioural intention [31, 32]. Cognitive absorption (a state of deep involvement with software) influences both perceived usefulness and perceived ease of use [33, 34]. Technical support has a direct significant effect on both perceived ease of use and perceived usefulness [35]. Computer anxiety (the degree of an individual’s concern or even fear, when she/he is faced by the possibility of using computers) has direct effects on perceived ease of use [25]. Lastly, the system’s features have a positive effect on the perceived usefulness of e-learning technology [30, 34].

In addition, the literature presents gender as a moderator of Technology Acceptance Model in the e-learning technology context. In a sample of Taiwanese workers the results show some differences in relation to gender [29] (see next section).

### E-Learning and gender

There is a controversial debate about the role of gender in education with regard to the similarities and differences between men and women, and their pedagogical implications. This debate started in the 70s when the issue of differentiated instruction and the ideal of gender equality was raised. Beyond the controversy, and the possible desirability of an adapted education for males and females, over the years research has been noting differences between men and women influencing pedagogical issues. For example, the existence of differential attendance rates between male and female students [36], gender differences in communicative style and approach to study [37], gender effects in levels of achievement motivations for subjects [38], whether the impact of social integration on subsequent institutional commitment is conditioned by gender [39] or the gender gap in study abroad participation [40]. In relation to e-learning, gender equity is a factor to be considered in designing courses. In fact, Garland and Martin [41], based on a sample of students enrolled in on-line courses, found that there was a difference in the learning style of the on-line student and the student in the face-to-face course, and that gender was a factor in the association between learning style and student engagement. According to these authors, the last finding supports the need for including gender equity in building and designing courses and programmes. E-learning valuation and satisfaction are greater among male students than female students [42]. Nevertheless, some research studies suggest that gender has no effect on satisfaction or attitudes towards e-learning [43, 44], or on teaching results [45, 46]. In addition, Cuadrado-García *et al*. [43] evaluated the existence of significant differences in relation to gender in the assessment and use of e-learning activities by students of two European universities. They found that there are few differences between male and female students in their satisfaction about e-learning activities. Furthermore, the study of Hung *et al*. [44] validated a multidimensional instrument for college students’ readiness for e-learning in Taiwan. The instrument used had five dimensions: self-directed learning, motivation for learning, computer/Internet self-efficacy, learner control, and on-line communication self-efficacy. Their results revealed that gender made no statistical differences along the dimensions of on-line learning readiness. Likewise, the research of Chu [45], based on adult e-learning students, shows that there are similarities between women and seniors beyond gender-related differences. Moreover, Kay and Knaack [46] showed no gender differences between males and females with respect to academic performance in secondary students. Female students even score e-learning courses higher on average than male students [9]. From a psychological standpoint, females are oriented to engagement, contact and taking care of other people, so they are more inclined towards human relationships. However, males are oriented to separation and abstract thinking, which predispose them towards personal achievement and subordinate relationships. Moreover, brain research has supported the existence of differences in brain structure between men and women at birth, without prejudice to a response to the influences of their environment [47].

In the field of Web-based learning, the lack of such gender-related research is clear. However, we found some papers that focus on gender differences and their consequences. Starting with the basics—use of computers—there are studies that find significant differences in the attitude to computers [48], and in perceived self-efficacy regarding completion of tasks. Males feel safer than females in the use of computers. Going one step further, focusing on Internet, studies show how, due to differences by gender in Internet usage and preferences by tasks, men and women perceive and use the Internet in a different way [49]. In this sense, each gender uses technology differently. Males tend to use the Internet and the Web to find information, while females normally use the Web to communicate to others [8]. In addition, male students feel more at ease with e-learning than female students [50]. What is more, males’ e-learning satisfaction was higher than that of females in a northern Taiwan university. Specifically, Lu and Chiou [51], based on five hundred and twenty-two university students from Taiwan, analysed satisfaction with e-learning systems. Their results showed that two contingent variables, gender and job status, significantly influenced the students' satisfaction with the e-learning system. However, in a study carried out in Sweden, women were more positive towards e-learning than men [52]. This was confirmed by a different study that took place in Taiwan [53].

The evidence about the effect of gender on the acceptance of information technology is not conclusive [54]. The results of previous studies show conflicting evidence in relation to whether gender affects or not the likelihood of using a computer system. For example, some results indicate the existence of such effects [55], and on the contrary, other findings indicate that these effects may disappear, especially in a young population [56]. Also, in Web environments clear evidence on gender-related effects does not appear. A study reports that there are not statistically significant differences between men and women in the process of adopting a particular Web technology [57]. In contrast, there is previous evidence of gender-related effects in the context of the adoption of e-learning [29]. Particularly, men's perceptions on perceived usefulness, perceived ease of use and behavioural intention to use e-learning are higher than women’s perceptions. In addition, perceived usefulness influences behavioural intention to use e-learning more strongly for men than for women. Likewise, perceived ease of use influences the perceived usefulness of e-learning with more force in women than in men [29]. Similar results were obtained by other authors [58], they found significant differences between men and women in the levels of behavioural intention to use the e-learning platform of a Spanish university. Furthermore, they showed that gender moderated the relationship between perceived usefulness and perceived ease of use. Similarly, a previous article [59] indicates that gender moderates the relationship between perceived usefulness and behavioural intention to use an e-learning platform in Lebanon. Also, other researchers [60] show that gender variable generates significant differences in relation to the usage of e-learning platforms. More specifically, they found that women use these platforms with greater frequency and intensity than men. According to this idea, they conclude that male and female students have a different behaviour for using e-learning platforms. Moreover, another study [61] indicates that the gender variable influences the behaviour of online learning in Taiwan. Considering the controversy explained above and the relevance of a reproduction in an ethnically distinct sample and based on a previous study [29], the following hypotheses are proposed:

**H0a:** Statistically significant differences between men and women exist in the scores of adoption of e-learning variables.

**H0b:** Statistically significant differences between men and women exist in the relationships between variables of the adoption of e-learning.

## Materials and Methods

### Research Model

**Fig 1:**
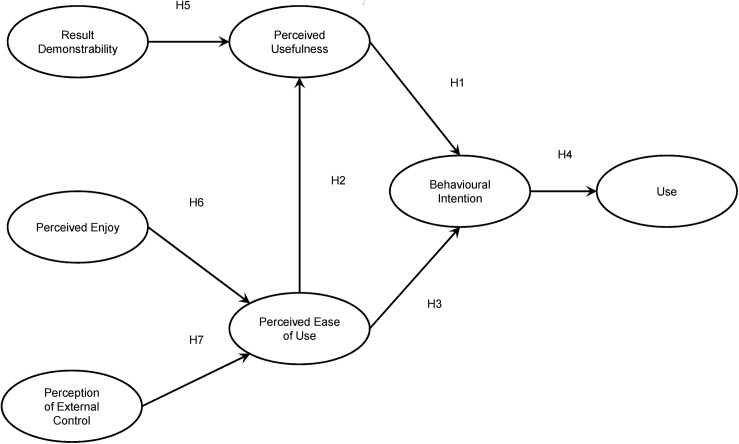
Model proposed.

We propose a model based on the basic Technology Acceptance Model, which relates the constructs perceived usefulness (PU), perceived ease of use (PEOU), and behavioural intention (BI). It also includes the effect of behavioural intention on use of the e-Learning Platform (USE). This basic Technology Acceptance Model is enriched with three antecedents, firstly shows that result demonstrability (RES—tangibility of the results of using the innovation) precedes perceived usefulness, and, secondly, perception of external control (PCE) and perceived enjoyment (ENJ) precede perceived ease of use. The proposed research model including 7 hypotheses is shown in Fig 1.

The Technology Acceptance Model has been utilised positively in the framework of e-learning [23]. Specifically, the results suggest that undergraduates employ an e-Learning Platform, if they recognise it as useful and easy to use [62]. Earlier, it has been specified that perceived ease of use and perceived usefulness are the key reasons acting on the disposition of university undergraduates to use e-learning [35]. Similarly, it has been confirmed that perceived usefulness straightaway affects the student's purpose to keep on using e-learning [63]. Bearing in mind the relevance of a reproduction in a sample which is ethnically diverse from those previously studied, and based on these preceding articles, the subsequent hypotheses are proposed:

**H1:** There is a positive relationship between perceived usefulness and behavioural intention in the adoption of e-learning.

**H2:** There is a positive relationship between perceived ease of use and perceived usefulness in the adoption of e-learning.

**H3:** There is a positive relationship between perceived ease of use and behavioural intention in the adoption of e-learning.

**H4:** There is a positive relationship between behavioural intention and use in the adoption of e-learning.

It is proposed that result demonstrability is an antecedent of perceived usefulness [12]. Moreover, it is proposed that perception of external control is an element that conditions perceived ease of use [12]. Perceived enjoyment is defined as the degree to which the activity of using an information technology is perceived as pleasant by itself, apart from the intrinsic instrumental value of the technology [64]. It is proposed that perceived enjoyment is positively related to perceived ease of use [12]. In the context of e-learning environments, another study [64] reported that perceived enjoyment directly affects perceived ease of use. Based on these results, the following second group of hypotheses is proposed:

**H5:** There is a positive relationship between result demonstrability and perceived usefulness in the adoption of e-learning.

**H6:** There is a positive relationship between perception of external control and perceived ease of use in the adoption of e-learning.

**H7:** There is a positive relationship between perceived enjoyment and perceived usefulness in the adoption of e-learning.

### Methodology

Data were obtained from a non-random sampling method. They were collected in Spain and Chile through an online questionnaire from May to July 2010.

Spain and Chile can be considered countries with a great access to information technologies. In fact, according to the latest Information and Communication Technology Development Index published in a total of 166 economies, Spain ranks 17th in Europe and 28th in the world, and Chile ranks second in Latin America and 56th in the world [65]. On the other hand, these two countries are among the 34 members of the Organisation for Economic Co-operation and Development (OECD). According to the latest data from the same organisation and in relation to higher education, it is impossible to say that there is a considerable gap between Spain and Chile [66]. In fact, the entry rates into tertiary education in Chile (34%) and in Spain (45%) are below the OECD average (48%). What is more, in relation to the total expenditure on educational institutions as a percentage of GDP, Chile (7%) is above and Spain (5%) is below the OECD average (6%). However, regarding the average earning's advantage for individuals who have had tertiary education; Spain ranks 25 out of 33, and Chile 1 out of 33.

The online survey was filled out by students of a University from the south of Spain and of a University from the north of Chile. Spanish respondents (230 students) were taking courses in the areas of Marketing and Business Management using an e-learning platform WebCT as a support of face-to-face courses (blended learning). WebCT (Course Tools) is at this time owned by Blackboard, an online proprietary virtual learning environment system that is licensed to colleges and universities used for e-learning. Trainers can add tools, such as discussion boards, mail systems, and live chat, along with content including documents and web pages. WebCT was the world's first broadly popular course management system for higher education. Nowadays, it is used by over 10 million students in 80 countries.

The Chilean respondents (159 students) were enrolled in courses of engineering using an e-learning platform ClaroLine as support of face-to-face courses (blended learning). Claroline is a collective eLearning platform distributed under the GPL Open Source license. It permits many organisations worldwide to generate and manage courses and cooperation sites over the web. Claroline is used in more than 100 countries and is available in 35 languages. This platform is the winner project of the 2007 UNESCO—King Hamad Bin Isa Al-Khalifa Prize for the Use of ICT in Education. Claroline integrates a big worldwide community.

Both platforms make available a comprehensive set of implements for subject planning, transfer, and organisation. Teachers have altogether the learning instruments they require to organise subjects and lessons tasks. Avoiding some particular use of the learning management system from a specific instructor, the use of the platforms by the group of teachers participating in the study in both universities was quite similar, and was a complement to traditional face-to-face lectures.

The exclusion of invalid questionnaires gave a sample size of 389 students, 201 males and 188 females (see Table 1).

**Table 1:** Descriptive statistics of the sample.

	Gender		
Country	Male (%)	Female (%)	Total (%)
Chile	116 (57.7)	43 (22.9)	159 (40.9)
Spain	85 (42.3)	145 (77.1)	230 (59.1)
Total (%)	201 (51.7)	188 (48.3)	389(100.0)

The average age of the interviewees was 23.12 years old and they had been enrolled for 4.03 years (on average) in tertiary education. So, they are students with extensive experience as college students. In particular, the average age of the male and female interviewees was 22.70 (SD = 5.37) and 23.44 (SD = 4.30) years-old, respectively. On the other hand, the average years of college of male and female respondents was 3.67 years (SD = 2.14) and 4.38 years (SD = 2.13), correspondingly. The population size was 1290 students from 2nd to 4th year in two faculties from both universities. The students were personally interviewed, in different classrooms, at the end of the term.

The scales applied have been verified in preceding studies [12, 28]. In particular, the scales used in this study have been taken without changes from a previous publication [28]. The survey was presented in Spanish. Previously, the questionnaire was translated from English to Spanish and after that back to English to guarantee correspondence in the translation, then a pre-test was conducted with fifty tertiary students. The surveys were carried out by personal interviews in order to collect instant feedback about the meaning of sentences. Some minor adaptations were made to make the Spanish language clear to Chilean speakers.

The partial least squares approach is a type of structural equation modelling that was used to test the research model proposed [67, 68]. Initially, this model was validated for the whole sample (389 cases). Then the sample was separated into two sets: males and females. A t-test was used to analyse if gender differences exist in the different constructs of the model. As the variables did not meet the requirement of normality, nonparametric techniques, specifically the Mann-Whitney test, were applied to corroborate the t-test results. Multi-group partial least squares analysis was then run to compare the differences between groups. SmartPLS software was used for this analysis [69].

### Ethics statement

Participation in the study was voluntary. All study participants were informed about the anonymity and confidentiality of their responses; the online platform to request the answers (www.limesurvey.org) was set to maintain the data anonymous. According to standard socio-economic studies, no ethical concerns are involved other than preserving the anonymity of participants. This procedure was approved by both the Head of the School of Engineering Systems and Computing of the Catholic University of Chile and the Head of the School of Business of the University of Seville. All study participants belonged to these institutions. At that time, an official IRB (Institutional Review Board) committee had not been established at these Universities.

## Results

The results of the descriptive statistics are shown in Table 2. The scale used is a 5-point Likert type, except the variable USE that is measured in minutes per week. SPSS software was used for this test.

**Table 2:** Descriptive statistics and T Test.

Latent Variable	Gender	Mean (SD)	T test (Sig.)
PU	Male	3.85 (.85)	-1.327
	Female	3.95 (.70)	(.183)
PEOU	Male	4.13 (.82)	-1.327
	Female	4.30 (.65)	(.023)
PCE	Male	3.89 (.63)	-1.327
	Female	4.05 (.60)	(.011)
ENJ	Male	3.22 (.94)	-1.327
	Female	3.28 (.72)	(.546)
RES	Male	3.43 (.89)	-1.327
	Female	3.57 (.74)	(.077)
BI	Male	4.04 (.83)	-1.327
	Female	4.20 (.80)	(.060)
USE	Male	56.77 (55.23)	-1.327
	Female	84.77 (101.08)	(.001)

As you can see in Table 2, the t-test results indicated statistically significant differences between the scores of men and women in some variables: use, perceived ease of use, perception of external control and behavioural intention. The Mann Whitney non-parametric estimates provided similar results. Therefore, hypothesis H0a (Statistically significant differences between men and women exist in the scores of adoption of e-learning variables) is partially accepted. Only in four variables the mean scores of females were significantly higher than the mean scores of males.

### Results of the measurement model

A partial least squares approach is applied to two models: the measurement model and the structural model. As a previous step in the structural analysis, the analysis of the reliability and validity of the measurement model is required. Exploratory factor analysis (using varimax rotation and principal components) was applied and every individual item was grouped inside the corresponding construct, achieving an explained total variance of 72.9%.

With regard to the content validity, this is based on the theoretical and empirical evidence supported by the measurement instruments used. Specifically, the content validity of Technology Acceptance Model scales is based on the rigorous procedure in the development of the scales included in the questionnaire. Thus, in the literature review, theoretical, conceptual and empirical aspects were considered. Furthermore, the pre-test provides a guarantee to support that content validity.

As shown in Table 3, reliability was evaluated by examining individual loads or simple correlations of the measures with their respective latent variables (≥ 0.7 were accepted). Cronbach's alpha coefficient was used as the reliability index of the latent variables. Moreover, composite reliability was computed. The convergent validity of the latent variables was evaluated by inspecting the average variance extracted (AVE), (> 0.5 were accepted).

**Table 3:** Cronbach’s Alpha, AVE, Composite Reliability and Factor Loadings.

Items		All	Males	Females
Behavioural Intentions(BI)	AVE	.72	.74	.69
	Composite Reliability	.88	.89	.87
	Cronbach's Alpha	.80	.82	.78
BI1		.82	.83	.82
BI2		.89	.90	.88
BI3		.82	.84	.80
Perception of External Control (PCE)	AVE	.69	.69	.69
	Composite Reliability	.87	.87	.87
	Cronbach's Alpha	.77	.77	.78
PCE1		.80	.80	.81
PCE2		.83	.83	.82
PCE3		.86	.86	.86
Perceived Ease of Use (PEOU)	AVE	.71	.73	.68
	Composite Reliability	.91	.92	.89
	Cronbach's Alpha	.86	.88	.84
PEOU1		.88	.88	.87
PEOU2		.85	.86	.82
PEOU3		.89	.89	.88
PEOU4		.75	.78	.73
Perceived Usefulness(PU)	AVE	.73	.75	.71
	Composite Reliability	.92	.92	.91
	Cronbach's Alpha	.88	.89	.86
PU1		.87	.88	.86
PU2		.87	.89	.82
PU3		.86	.88	.83
PU4		.82	.80	.85
Perceived Enjoy (ENJ)	AVE	.79	.81	.75
	Composite Reliability	.92	.93	.90
	Cronbach's Alpha	.88	.89	.85
ENJ1		.88	.88	.85
ENJ2		.93	.93	.94
ENJ3		.86	.89	.80
Result Demonstrability (RES)	AVE	.78	.83	.71
	Composite Reliability	.92	.94	.88
	Cronbach's Alpha	.86	.90	.80
RES1		.90	.93	.83
RES2		.87	.90	.82
RES3		.89	.90	.87
USE				
USE		N.A.	N.A.	N.A.

To verify the discriminant validity of the latent variables, a partial least squares model was carried out to examine their measurement properties. In addition, the Fornell-Larcker criterion indicated that discriminant validity is established if a latent variable accounts for more variance in its associated indicator variables than it shares with other constructs in the same model. According to this idea, it was examined whether the square root of the AVE of each variable was higher than the correlations with other variables. Table 4 shows the results for the whole sample.

**Table 4:** Squared correlations for the complete sample and AVE.

	BI	ENJ	PCE	PEOU	PU	RES	USE
BI	.85						
ENJ	.33	.89					
PCE	.38	.22	.83				
PEOU	.42	.29	.61	.84			
PU	.42	.44	.39	.42	.86		
RES	.38	.49	.23	.34	.44	.89	
USE	.18	.08	.06	.12	.13	.16	1.00

### Results of the structural model

After examining the measurement model using partial least squares, the relations between the constructs were addressed. The hypotheses were verified by exploring the path coefficients. A bootstrapping of 500 sub-samples was computed to verify the statistical significance of each path. The variance explained (R-squared) in the endogenous latent variables and the p-values of regression coefficients (F-test) work as indicators of the explanatory power of the model.

The outcomes of the multi-group analysis for the model with the groups of males and females are shown in Table 5. Based on these results, hypotheses H1, H2, H3, H4, H5, H6 and H7 are accepted because all the relationships hypothesised are statistically significant.

**Table 5:** Path coefficients.

Path	Men (Sig.)	Females (Sig.)	|Men-Females|	Henseler’ p-value (Sig.)	T-value	Parametricp value (Sig.)
BI ->USE	.20 (**)	.16 (*)	.04	.37 (n.s.)	.37	.71 (n.s.)
ENJ->PEOU	.19 (**)	.11 (n.s.)	.09	.18 (n.s.)	.93	.35 (n.s.)
PCE->PEOU	.53 (***)	.64 (***)	.10	.86 (n.s.)	1.09	.28 (n.s.)
PEOU->BI	.32 (***)	.26 (***)	.05	.32 (n.s.)	.45	.65 (n.s.)
PEOU->PU	.37 (***)	.19 (**)	.18	.06 (n.s.)	1.60	.11 (n.s.)
PU->BI	.26 (**)	.34 (***)	.08	.75 (n.s.)	.70	.49 (n.s.)
RES->PU	.33 (***)	.33 (***)	.01	.49 (n.s.)	.05	.96 (n.s.)

Statistical significance

* p<0.05

**p<0.01

***p<0.001

n.s. non-significant.

**Fig 2:**
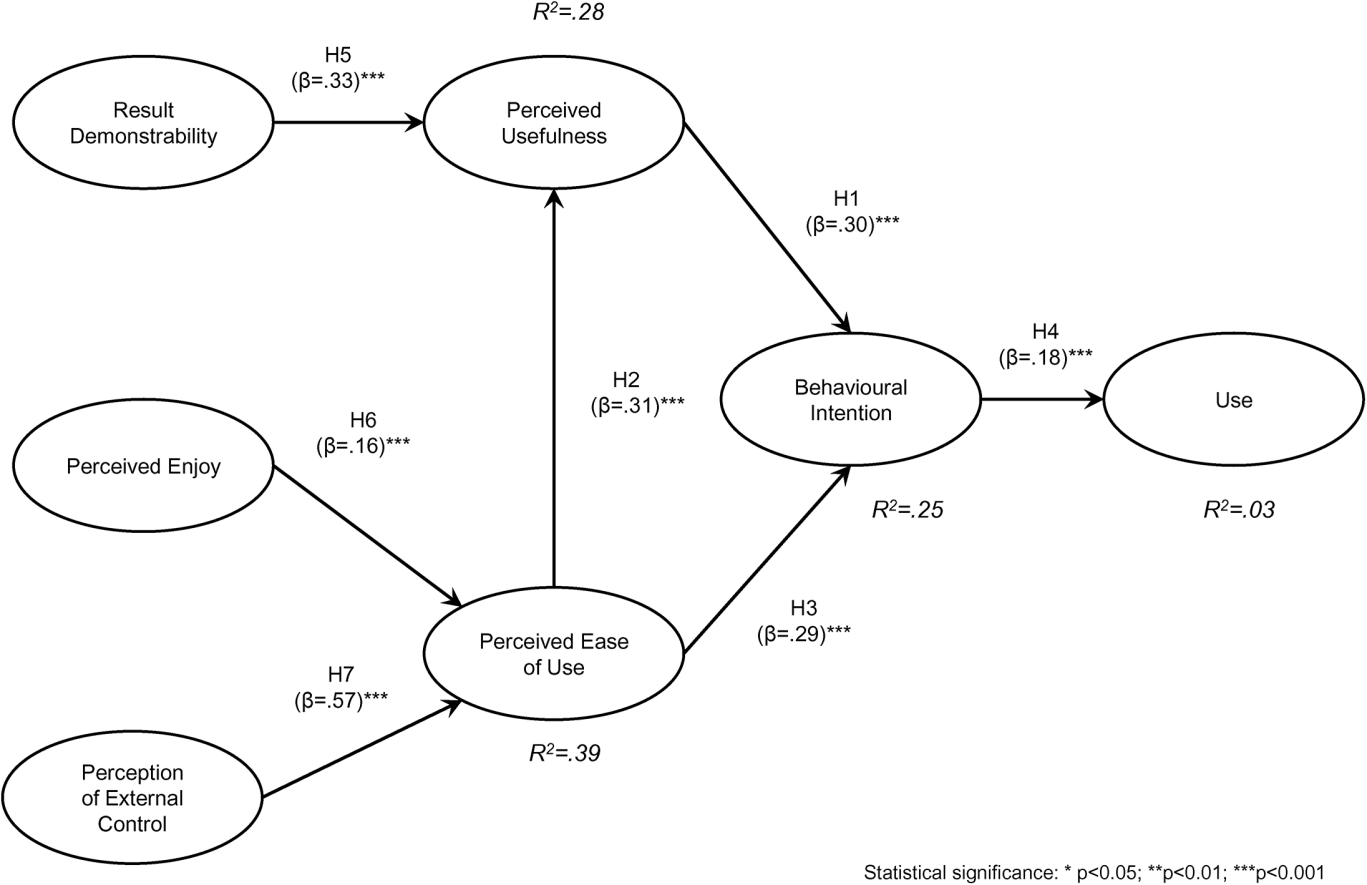
Partial Least Squares results for the whole sample.

From this table we cannot accept hypothesis H0b (Statistically significant differences between men and women exist in relationships between variables of the adoption of e-learning), because statistically significant differences between men and women do not exist in the relationships between variables of the adoption of e-learning in our model. Fig 2 shows the result for the model considering the whole sample.

## Discussion

To sum up, we highlight this study’s main four contributions. Firstly, a version of the Technology Acceptance Model that includes elements of Technology Acceptance Model 2 and Technology Acceptance Model 3 has been successfully used to explain the process of the adoption of e-learning in tertiary education in two universities from two different countries. This means you can use a tested tool adapted from other areas of technology in the field of virtual education platforms, helping to improve these educational techniques that will undoubtedly gain weight in college learning.

Secondly, the finding in the use of e-learning platform of a strong and significant relationship between perception of external control and perceived ease of use is noteworthy. This has implications for the design of these platforms in relation to the control and resources given to users. This can be interpreted as an indication that users of these platforms prefer having control over the system. It is likely that students prefer to customise the platform environment. These results support the study [70] who found that for males it is more efficient to present information using graphics and, in the case of females, the designers of educational software should avoid detailed and colourful artistic drawings. However, in order to customise the interfaces and improve the control over the system, it is necessary for users to have a certain knowledge and skills about the use of digital tools [71]. When these skills are increased, the perception of the ease of use of online learning tools is improved too. In this sense, the results of this paper contribute to explaining those obtained from previous investigations. For example, some authors [72] found a very favourable opinion from the majority of students regarding the use of virtual learning platforms. Nonetheless, there is a small group of students characterised by scant skills in this new environment, which makes them reluctant towards its use. In the same way, other authors [73] discovered some groups of students dissatisfied with the use of information technology in higher education. Also, another study [74] arrives at similar conclusions in the field of university professors.

Thirdly, according to the previous literature, a significant relationship between perceived enjoyment and perceived ease of use and between result demonstrability and perceived usefulness is found. If students conceive the use of the e-learning platform as fun and enjoyable they show a higher perceived ease of use. An additional consequence is that students who communicate the possibilities of the e-learning platform to others give it more value. These results are according to the previous conclusions [74] in the context of university teachers: students will use technological resources if these are visible.

Last but not least, the study indicates a few statistically significant differences between males and females when adopting an e-learning platform according to the model tested, so hypothesis H0a is partially supported. Four constructs show differences by gender: perceived ease of use, perception of external control, behavioural intention, and use. In all cases the scores obtained by females are higher than those obtained by males. The fact that the use and behavioural intention of e-learning platforms are higher in females stands out, and this is good news as it shows the disappearance of the traditional gap between men and women with regard to the adoption of new technologies, at least for this sample of college students. However, multi-group analysis does not support hypothesis H0b and no significant differences have been found between men and women in our model’s relationships. But the results show some slight differences between both genders. The relationship between (1) perceived ease of use and behavioural intention and (2) perceived ease of use and perceived usefulness is a bit stronger among the males of the sample. What is more, the same occurs in the relationship between perceived enjoyment and perceived ease of use. By contrast, among female students the relationship between perception of external control and perceived ease of use is stronger. This seems to indicate that this group of students value more the perception of a greater control of the virtual learning platform. The relationship between perceived usefulness and behavioural intention is also slightly stronger among the women of the study. According to the results of the multi-group partial least squares analysis, the women of the sample have a stronger perception of external control than the men using the e-learning platform. Although the results of other authors [29] report different reactions to gender in the adoption of e-learning platforms, the conclusions of this study are closer to the ideas of other researchers [56, 57]. Consistent with a previous article [56], we believe that analysing a sample of university students (not employees) is a key point to explaining this result. Both male and female students have equal educational technology in the classroom. Often, they have similar previous training, especially in the higher courses and have a very similar experience as learners. In spite of this, some differences appear between both groups. This may be one reason why gender inequalities regarding the perception of new technologies that often occur in other areas do not appear so intensely among higher education students [28]. Further research is necessary to continue the work on this topic.

The main implications of this study are that users of these e-learning platforms wish to manipulate the system. It is expected that undergraduates prefer to adapt the platform setting. If students consider the use of the e-learning platform as cool and pleasant, they exhibit a higher perceived ease of use. A supplementary outcome is that undergraduates who transmit the potentials of the e-learning platform to others give it more worth. It is outstanding that the use and behavioural intention of e-learning platforms are greater among females, and this is valuable information because it displays the evaporation of the conventional disparity between males and females with regard to the adoption of information technologies, at least for this sample of college students. As a consequence, the adaptation of e-learning platforms by gender does not seem to be necessary for tertiary students.

This study has some limitations that may guide future work. First, the validation of the results requires a larger sample of individuals. Second, the use of a non-random sampling method within a single organisation limits the generalisation of findings. Third, the study is cross-sectional; a longitudinal study would be advisable to compare the different stages of the adoption of e-learning. Finally, it would be useful to incorporate more students from other knowledge areas different from marketing, business and engineering, such as other social sciences and humanities. Also, including students in early degree courses with less experience as learners and users of these e-learning platforms may yield more complete and detailed studies.

## Supporting Information

S1 Dataset (XLS)

S1 Questionnaire (DOCX)

S2 Questionnaire (DOCX)
