# Supplementary material for: Gender and Acceptance of E-Learning: A Multi-Group Analysis Based on a Structural Equation Model among College Students in Chile and Spain
Source: PLoS One. 2015 Oct 14;10(10):e0140460. doi: 10.1371/journal.pone.0140460 (PMC4605762; doi:10.1371/journal.pone.0140460)
Supplement: S1 Questionnaire — (DOCX) [file pone.0140460.s002.docx]

**Cuestionario**

Estimado estudiante estamos analizando algunos aspectos de la plataforma de enseñanza virtual que usa en algunas asignaturas de sus estudios. Por favor, responda las siguientes cuestiones para mejorar el sistema.

Indique el valor que más se ajuste a su opinión. La escala de 5 puntos significa (1: fuerte desacuerdo, 2: algo en desacuerdo, 3: neutro (ni acuerdo ni desacuerdo), 4: algo de acuerdo, 5: muy de acuerdo)

| **Utilidad percibida (PU)** |  |
| --- | --- |
| PU1 Usar el sistema de enseñanza virtual mejora mi desempeño en mis estudios | 1 2 3 4 5 |
| PU2 Usar el sistema de enseñanza virtual en mis estudios mejora mi productividad | 1 2 3 4 5 |
| PU3 Usar el sistema de enseñanza virtual refuerza mi efectividad en mis estudios | 1 2 3 4 5 |
| PU4 Encuentro que el sistema de enseñanza virtual es útil para mis estudios | 1 2 3 4 5 |
|  | |
| **Facilidad de uso percibida (PEOU)** |  |
| PEOU1 Mi interacción con el sistema de enseñanza virtual es clara y comprensible | 1 2 3 4 5 |
| PEOU2 Interactuar con el sistema de enseñanza virtual no requiere mucho esfuerzo mental | 1 2 3 4 5 |
| PEOU3 Encuentro el sistema de enseñanza virtual fácil de usar | 1 2 3 4 5 |
| PEOU4 Encuentro fácil que el sistema de enseñanza virtual haga lo que yo le pido | 1 2 3 4 5 |
|  | |
| **Percepciones de control externo (PEC)** |  |
| PEC1 Tengo el control sobre el uso del sistema | 1 2 3 4 5 |
| PEC2 Tengo los recursos necesarios para usar el sistema | 1 2 3 4 5 |
| PEC3 Dados los recursos, oportunidades y conocimiento que requiere el uso del sistema, sería fácil para mí usar el sistema. | 1 2 3 4 5 |
|  | |
| **Disfrute percibido (ENJ)** |  |
| ENJ1 Encuentro el uso del sistema de enseñanza virtual divertido | 1 2 3 4 5 |
| ENJ2 El proceso actual de usar el sistema de enseñanza virtual es agradable | 1 2 3 4 5 |
| ENJ3 Me lo paso bien usando el sistema | 1 2 3 4 5 |
|  |  |
| **Demostrabilidad de los resultados (RES)** |  |
| RES1 No tengo problema en contarle a otros los resultados de usar el sistema de enseñanza virtual | 1 2 3 4 5 |
| RES2 Creo que comunicaría a otros las consecuencias de usar el sistema de enseñanza virtual | 1 2 3 4 5 |
| RES3 Creo que los resultados de usar el sistema de enseñanza virtual son evidentes | 1 2 3 4 5 |
|  |  |
| **Intención de comportamiento (BI)** |  |
| BI1 Asumiendo que tuviera acceso al sistema de enseñanza virtual, intento usarlo | 1 2 3 4 5 |
| BI2 Dado que tengo acceso al sistema de enseñanza virtual, pronostico que lo usaré | 1 2 3 4 5 |
| BI3 Tengo planeado usar el sistema de enseñanza virtual en los próximos <n> meses | 1 2 3 4 5 |
|  |  |
| **Use (USE)** |  |
| USE1 De media, ¿cuánto tiempo se lleva usando el sistema de enseñanza virtual cada día? (en minutos) |  |

Información personal (por objetivos estadísticos):

Usted es:

1. hombre

2. mujer

Su edad es: ___

*Muchas gracias por su cooperación*
