# Supplementary material for: Gender and Acceptance of E-Learning: A Multi-Group Analysis Based on a Structural Equation Model among College Students in Chile and Spain
Source: PLoS One. 2015 Oct 14;10(10):e0140460. doi: 10.1371/journal.pone.0140460 (PMC4605762; doi:10.1371/journal.pone.0140460)
Supplement: S2 Questionnaire — (DOCX) [file pone.0140460.s003.docx]

**Questionnaire**

Dear student, we are analyzing some aspects of virtual learning platform that you are using in some courses of studies. Please answer the following questions to improve the system.

Specify the value that best suits your opinion. The 5-point scale means the following (1: strongly disagree, 2: somewhat disagree, 3: neutral (neither agree nor disagree), 4: somewhat agree, 5: strongly agree)

| **Perceived Usefulness (PU)** |  |
| --- | --- |
| PU1 Using E-learning platform improves my performance in my studies | 1 2 3 4 5 |
| PU2 Using E-learning platform in my studies increases my productivity | 1 2 3 4 5 |
| PU3 Using E-learning platform enhances my effectiveness in my studies | 1 2 3 4 5 |
| PU4 I find E-learning platform to be useful in my job/studies | 1 2 3 4 5 |
|  | |
| **Perceived Ease of Use (PEOU)** |  |
| PEOU1 My interaction with E-learning platform is clear and understandable | 1 2 3 4 5 |
| PEOU2 Interacting with E-learning platform does not require a lot of my mental effort | 1 2 3 4 5 |
| PEOU3 I find E-learning platform to be easy to use | 1 2 3 4 5 |
| PEOU4 I find it easy to get E-learning platform to do what I want it to do | 1 2 3 4 5 |
|  | |
| **Perception of External Control (PEC)** |  |
| PEC 1 I have control over using the system | 1 2 3 4 5 |
| PEC 2 I have the resources necessary to use the system | 1 2 3 4 5 |
| PEC 3 Given the resources, opportunities and knowledge it takes to use the system, it is easy for me to use the system | 1 2 3 4 5 |
|  | |
| **Perceived Enjoy (ENJ)** |  |
| ENJ1 I find using E-learning platform to be enjoyable | 1 2 3 4 5 |
| ENJ2 The actual process of using E-learning platform is pleasant | 1 2 3 4 5 |
| ENJ3 I have fun using E-learning platform | 1 2 3 4 5 |
|  |  |
| **Result Demonstrability (RES)** |  |
| RES 1 I have no difficulty telling others about the results of using E- learning platform | 1 2 3 4 5 |
| RES 2 I believe I could communicate to others the consequences of using E-learning platform | 1 2 3 4 5 |
| RES 3 The results of using E-learning platform are apparent to me | 1 2 3 4 5 |
|  |  |
| **Behavioural Intentions (BI)** |  |
| BI1 Assuming I had access to E-learning platform, I intend to use it | 1 2 3 4 5 |
| BI2 Given that I had access to E-learning platform, I predict that I would use it | 1 2 3 4 5 |
| BI3 I plan to use E-learning platform in the next months | 1 2 3 4 5 |
|  |  |
| **Use (USE)** |  |
| USE1 On average, how much time do you spend on E-learning platform each day? (In minutes) |  |

Personal information (for statistical purposes):

You are:

1. man

2. women

Your age is: ___

*Thank you very much for your cooperation*
